# Comparison of Four Ground-Level PM_2.5_ Estimation Models Using PARASOL Aerosol Optical Depth Data from China

**DOI:** 10.3390/ijerph13020180

**Published:** 2016-01-30

**Authors:** Hong Guo, Tianhai Cheng, Xingfa Gu, Hao Chen, Ying Wang, Fengjie Zheng, Kunshen Xiang

**Affiliations:** State Key Laboratory of Remote Sensing Science, Institute of Remote Sensing and Digital Earth, Chinese Academy of Sciences, Beijing 100101, China; guohong@radi.ac.cn (H.G.); guxf@radi.ac.cn (X.G.); chenhao@radi.ac.cn (H.C.); wangying@radi.ac.cn (Y.W.); zhengfj@radi.ac.cn (F.Z.); sheva5070@163.com (K.X.)

**Keywords:** PM_2.5_ concentrations, fine-mode aerosol, polarized remote sensing, air quality monitoring, empirical models

## Abstract

Satellite remote sensing is of considerable importance for estimating ground-level PM_2.5_ concentrations to support environmental agencies monitoring air quality. However, most current studies have focused mainly on the application of MODIS aerosol optical depth (AOD) to predict PM_2.5_ concentrations, while PARASOL AOD, which is sensitive to fine-mode aerosols over land surfaces, has received little attention. In this study, we compared a linear regression model, a quadratic regression model, a power regression model and a logarithmic regression model, which were developed using PARASOL level 2 AOD collected in China from 18 January 2013 to 10 October 2013. We obtained R (correlation coefficient) values of 0.64, 0.63, 0.62, and 0.57 for the four models when they were cross validated with the observed values. Furthermore, after all the data were classified into six levels according to the Air Quality Index (AQI), a low level of statistical significance between the four empirical models was found when the ground-level PM_2.5_ concentrations were greater than 75 μg/m^3^. The maximum *R* value was 0.44 (for the logarithmic regression model and the power model), and the minimum *R* value was 0.28 (for the logarithmic regression model and the power model) when the PM_2.5_ concentrations were less than 75 μg/m^3^. We also discussed uncertainty sources and possible improvements.

## 1. Introduction

Numerous epidemiological studies have been conducted to determine the relationship between human health and fine particulate matter (PM_2.5_, particles with an aerodynamic diameter ≤ 2.5 μm), and have indicated that they are highly correlated; therefore, accurate and long-term PM_2.5_ data are very important for epidemiological studies [1,2,3,4]. Ground-level PM_2.5_ monitoring sites can provide accurate and real-time data. However, their spatial coverage is so limited that they are insufficient to capture the spatial variability of the PM_2.5_ concentrations for epidemiological studies [5,6].

With broad spatial coverage, satellite data provide a new way to supplement and expand ground monitoring networks to study the spatial variations of PM_2.5_ concentrations, particularly in suburban and rural areas. AOD data, which can be retrieved from satellite data, represent aerosols in the atmospheric column and can be used as a quantitative measurement of PM_2.5_ concentrations [7]. Indeed, many studies have exploited the quantitative relationship between satellite-derived AOD data and ground-level PM_2.5_ concentrations [8,9,10,11,12,13,14,15,16].

To date, most current studies have focused on developing empirical models based on ground-level PM_2.5_ concentrations and MODIS/MISR AOD data [17,18,19,20,21,22]. For example, Saunders *et al.* [23] improved the estimation of PM_2.5_ using Lagrangian MODIS AOD data, and the results showed an R-squared value of 0.51 for the ground-level observed PM_2.5_ and satellite-predicted PM_2.5_ concentrations. Van Donkelaar *et al.* [19] developed global exposure estimates of ambient PM_2.5_ mass and trends using PM_2.5_ concentrations inferred from multiple satellite instruments (SeaWIFS, MISR, and MODIS) and Chemistry Transport Model simulations (GEOS-Chem); their results indicated an *R* value of 0.81 for satellite-derived PM_2.5_ *versus* ground-level PM_2.5_ on a global scale. In addition, a vertical-and-relative humidity correcting method based on MODIS AOD and meteorological data were also used to estimate ground-level PM_2.5_ [8,13,20]. However, few studies have been devoted to developing empirical models based on ground-level PM_2.5_ concentrations and POLDER/PARASOL AOD data, which is sensitive to fine-mode aerosols over land surfaces [24,25,26,27,28,29,30,31]. Kacenelenbogen *et al.* [32] developed linear regression models between ground-level PM_2.5_ concentrations and POLDER-2 AOD data (at 440 nm) over France, and pointed out that the lack of higher PM_2.5_ concentrations in their data prevented them from studying air quality categories other than “good” or “moderate”.

With the rapid economic development during the recent 30 years, PM_2.5_ pollution has become a severe atmospheric environmental problem and caused widespread public concern in China, especially in the last 5 years. However, due to the high operational costs of ground monitoring stations, most cities in China lack continuous spatial and temporal ground-level PM_2.5_ data prior to 2013; therefore, it is difficult to assess the spatial and temporal variability of PM_2.5_ concentrations using ground-level PM_2.5_ data or satellite-based PM_2.5_ estimation models. Fortunately, we can access considerable ground-level PM_2.5_ data from the official website of the China National Environmental Monitoring Center (CNEMC) [33] since January 2013.

In comparison to other studies using total AOD data (e.g., MODIS AOD), the vertical-and-relative humidity correcting method does not work well for PARASOL AOD data. Therefore, the aims of this paper are: (1) to develop the best empirical models using PARASOL level 2 AOD and ground-level PM_2.5_ concentrations over China from 18 January 2013 to 10 October 2013; (2) to compare the spatial distribution of PM_2.5_ concentrations with the four developed models; (3) to validate the four developed models by comparing the predicted PM_2.5_ concentrations with the ground-level PM_2.5_ concentrations; and (4) to compare the PM_2.5_ concentrations of the four developed models using the AQI.

## 2. Materials and Methods

### 2.1. Parasol Aod

The PARASOL payload consists of a multi-spectral, multi-directional, and polarization-capable radiometer, which was launched onboard the ADEOS platforms on 18 December 2004. The PARASOL scientific mission started on 12 March 2005 and ended on 11 October 2013 [34,35,36]. Over land surfaces, the PARASOL aerosol operational retrieval is based on polarized measurements at 0.670 µm and 0.865 µm [24]. Contrary to the total radiances, the polarized reflectance of surfaces is small and fairly spectrally independent [37], and the atmospheric contribution is larger than the surface polarized reflectance. The aerosol models used in the land operational algorithm only consider aerosols in the accumulation mode (radii less than approximately 0.5 µm), and the contribution of the coarse mode is neglected. The refractive index is taken equal to 1.47–0.01i, which corresponds to a mean value for aerosols resulting from biomass burning or pollution events [38]. The surface contribution depends on the surface type, *i.e.*, bare soils or vegetated areas, and is estimated from a relationship using empirical coefficients adjusted for the different classes of land surfaces according to the main International Geosphere Biosphere Programme (IGBP) biotypes and the NDVI. The PARASOL operational algorithm has successfully provided 18.5 × 18.5 km^2^ resolution AOD (865 nm) data over land surfaces [24,39,40,41,42].

Several studies [43,44] have already validated the accuracy of PARASOL level 2 AOD by comparing PARASOL level 2 AOD against AERONET AOD. Fan *et al.* [43] compared PARASOL Level 2 AOD data (particles with radii less than or equal to 0.3 µm) over land surfaces against AERONET Level 1.0 AOD data from Beijing and Xianghe in China, and the results showed that the slope and correlation between the two data sets were both close to 1, and the AOD RMS (root mean square) was 0.03, which demonstrates the capability of PARASOL for determining the anthropogenic contribution of regional aerosols. In addition, Su *et al.* [44] compared PARASOL Level 2 AOD data (particles with radii less than or equal to 0.3 µm) over land surfaces against AERONET Level 1.5 AOD data from 14 sites in East Asia. These authors reported a good correlation (*R* ≈ 0.92) between the two data sets and demonstrated the remarkable sensitivity of PARASOL AOD to the smaller fraction of fine particles that mostly originated from anthropogenic sources. In this study, PARASOL level 2 AOD data collected from 18 January 2013 to 10 October 2013 over China were selected for our research.

### 2.2. Ground-Level PM_2.5_ Concentrations

**Figure 1:**
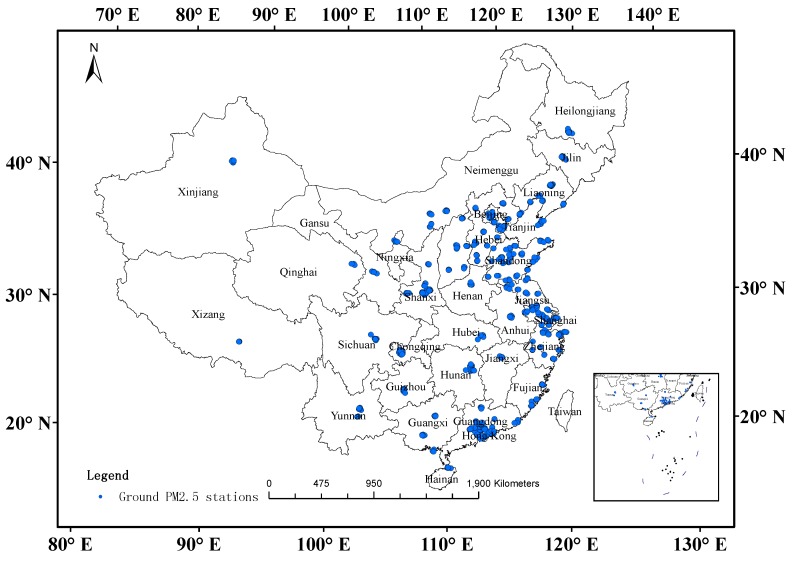
PM_2.5_ monitoring sites over mainland China.

The CNEMC had already established more than 600 PM_2.5_ monitoring sites in 119 cities across mainland China by October 2013, and the monitoring results of these PM_2.5_ concentrations have been public (Figure 1). Most of the PM_2.5_ monitoring sites are located in eastern China, while they are mainly located in the capital cities in mid-west China (e.g., Henan Province, Shanxi Province, Sichuan Province, Xinjiang Province, Xizang Province). The hourly average PM_2.5_ concentrations from 18 January 2013 to 10 October 2013 for 670 sites were downloaded from the official website of the CNEMC [33].

### 2.3. Data Processing

Because the time resolution of the ground-based PM_2.5_ data is hourly, and the spatial resolution of PARASOL level 2 AOD is about 18.5 km × 18.5 km, therefore, we collocated the ground-level PM_2.5_ concentrations with the PARASOL level 2 AOD when the distance and the time interval between the PARASOL level 2 AOD and ground-level PM_2.5_ data were within roughly 20 km and ±1 h, respectively. Finally, a total of 10,873 records were collected from 18 January 2013 to 10 October 2013 over mainland China. Due to the coarse spatial resolution (18.5 × 18.5 km^2^) of PARASOL level 2 AOD data, and the ground-level PM_2.5_ monitoring sites are often in close proximity within a city, there may be a case that one PARASOL level 2 AOD pixel corresponds to several different ground-level PM_2.5_ monitoring sites. In order to decrease the deviations, the average value of multiple ground-level PM_2.5_ concentrations was used to match the PARASOL level 2 AOD data, resulting in a total of 4339 matching records.

In addition, in order to facilitate comparison among the four developed models based on the PARASOL level 2 AOD and ground-level PM_2.5_ concentrations, we divided the entire dataset into model development subdata and model validation subdata; the subdata from January to August 2013 were used to construct the models (70.34% of the total data), while the subdata from September to October 2013 were used to verify the accuracy of the models (29.66% of the total data).

The number of records (N), geometric mean, the maximum and minimum of the two variables are presented for all days (Table 1). The mean PM_2.5_ concentrations and the PARASOL level 2 AOD data used for model development are 56.11 μg/m^3^ and 0.11, respectively, while the mean PM_2.5_ concentrations and the PARASOL level 2 AOD data used for model validation are 57.43 μg/m^3^ and 0.12, respectively. The Minimum of the PM_2.5_ concentrations and is 3 μg/m^3^, and the Minimum of the PARASOL level 2 AOD data is 0.002 for model development and validation, respectively.

**Table 1:** Summary statistics of PM_2.5_ concentrations and PARASOL level 2 AOD data.

Type	Parameter	*N*	Mean	Minimum	Maximum
Model Development	PM2.5 (μg/m^3^)	3052	56.11	3	1000
	AOD	3052	0.11	0.002	0.91
Model Validation	PM2.5 (μg/m^3^)	1287	57.43	3	562
	AOD	1287	0.12	0.002	0.59

### 2.4. Empirical Models

Empirical models are constructed to predict ground-level PM_2.5_ concentrations owing to only two parameters of PARASOL AOD and ground-level PM_2.5_ concentrations. Based on the analysis of eleven empirical models, a linear regression model, a quadratic regression model, a power regression model, and a logarithmic regression model were selected to predict ground-level PM_2.5_ concentrations. The SPSS 21.0 for Windows program (IBM, Stanford, CA, USA) was applied to all of the statistical analyses conducted in this research.

## 3. Results

### 3.1. Models Construction

**Figure 2:**
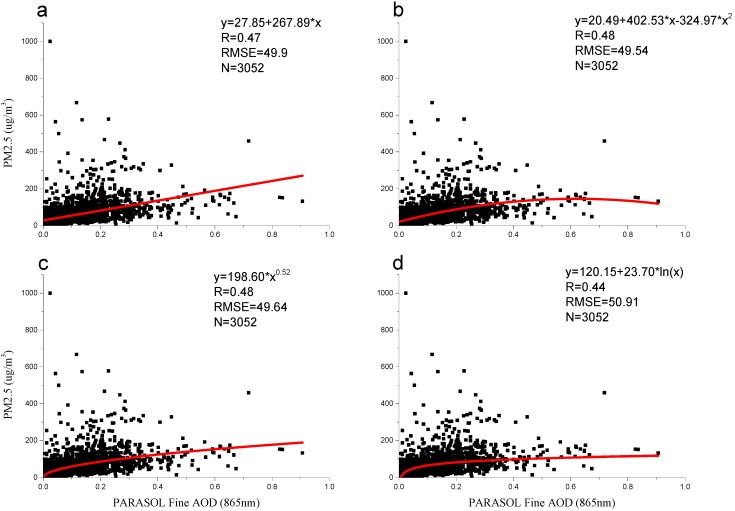
The four regression models based on PM_2.5_ concentrations and PARASOL AOD data (*p* < 0.05) (**a**) the linear regression model; (**b**) the quadratic regression model; (**c**) the power regression model; and (**d**) the logarithmic regression model.

According to the model development subdata, a linear regression model, a quadratic regression model, a power regression model, and a logarithmic regression model were developed (Figure 2). A comparison of the results indicated that the quadratic model and the power model were optimal due to their *R* values of 0.48, and their RMSE (root mean square error) values of 49.54 μg/m^3^ and 49.64 μg/m^3^, respectively. These models were followed by the linear model, with an *R* value of 0.47 and an RMSE value of 49.9 μg/m^3^. Finally, the logarithmic regression model performed the worst, indicated by the relatively low R value of 0.44 and the high RMSE value of 50.91 μg/m^3^. Although the R values and the RMSE values differed, the difference between the four models was small. The linear regression model agreed with the findings of Kacenelenbogen *et al.* [32], which obtained an *R* value of 0.55 using POLDER-2 AOD data (at 440 nm) to develop a linear regression model for France. However, although the linear regression model, the quadratic regression model, and the logarithmic regression model were similar to the research conducted by Wang *et al.* [22], the results were considerably different, *i.e.*, the R-square values they obtained for the linear regression model (0.75), the quadratic regression model (0.818), and the logarithmic regression model (0.629) were much higher than ours. The difference may be caused by the use of PARASOL AOD in our research instead of MODIS AOD used by Wang *et al.* [22].

### 3.2. The Spatial Distribution of PM2.5 Concentrations

**Figure 3:**
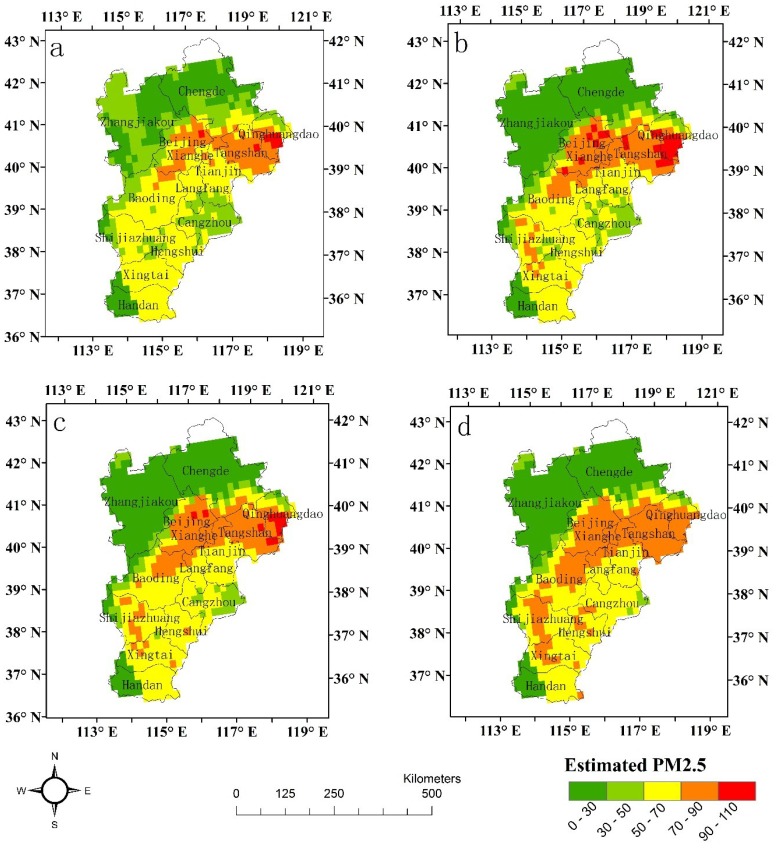
The PM_2.5_ spatial distribution based on the four models 2 April 2013) (**a**) the linear regression model; (**b**) the quadratic regression model; (**c**) the power regression model; and (**d**) the logarithmic regression model.

There are generally two orbits of PARASOL swaths over China daily. However, valid PARASOL level 2 AOD data are infrequent in most provinces in China due to its AOD inversion algorithms and coarse spatial resolution. Because of sufficient data, PARASOL level 2 AOD from 2 April 2013 was selected to estimate the ground-level PM_2.5_ concentrations in the cities of Beijing city and Tianjin and Hebei Province based on the four models (Figure 3). The PM_2.5_ concentrations were blank in some districts because of the lack of PARASOL level 2 AOD data.

It was noticed that the PM_2.5_ distribution maps based on the four models show some consistency. The results showed that Beijing, Tangshan and Qinhuangdao had the highest PM_2.5_ concentrations (>70.0 μg/m^3^), while Zhangjiakou and Chengde had the lowest PM_2.5_ concentrations (≤30.0 μg/m^3^). In addition, all four models underestimated the ground-level PM_2.5_ concentrations for heavy pollution events, for which the maximum satellite-estimated PM_2.5_ concentrations were 96.4 μg/m^3^, 102.2 μg/m^3^, 97.8 μg/m^3^, and 87.9 μg/m^3^ in Figure 3a–d, respectively, while the maximum ground-level observed value was 151.0 μg/m^3^ (Beijing). Similar results have been reported in previous studies when MODIS AOD data were used to estimate ground-level PM_2.5_ concentrations for heavy pollution events [45,46]. It may because PARASOL and MODIS observations cannot distinguish the aerosol vertical profile and often are unable to detect pollution build up in stagnant inversion layers.

However, it was also obvious that the PM_2.5_ distribution maps based on the four models exhibited some differences. Figure 3a shows that the area with higher PM_2.5_ concentrations (>70 μg/m^3^) was the smallest, and the area with the lowest PM_2.5_ concentrations (≤30 μg/m^3^) was the largest among the four maps (Figure 3b), while the areas with PM_2.5_ concentrations between 50.01 μg/m^3^ and 70 μg/m^3^ in Figure 3c,d are larger than those in Figure 3a,b. Although the difference between Figure 3c,d is small on the map, it is easy to determine that the maximum satellite-estimated PM_2.5_ concentrations in Figure 3c are larger than those in Figure 3d, and the satellite-estimated PM_2.5_ values in Figure 3c are lower than those in Figure 3d for Cangzhou City.

In summary, although there was a difference between the ground-level PM_2.5_ concentrations and the satellite-derived PM_2.5_ concentrations based on the four models, the results showed similar spatial variation trends of the PM_2.5_ concentrations among the models.

### 3.3. Models Validation

**Figure 4:**
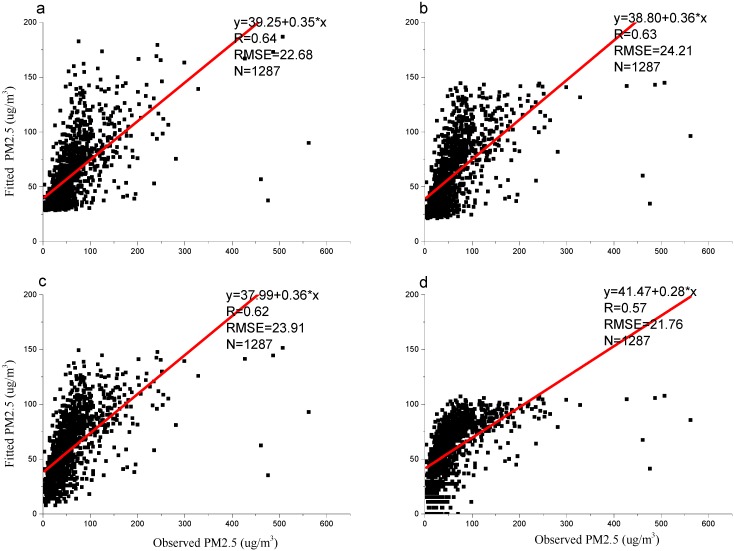
The validation of the four models (*p* < 0.05) (**a**) the linear regression model; (**b**) the quadratic regression model; (**c**) the power regression model, and (**d**) the logarithmic regression model.

A cross-validation approach was used to evaluate the ability of the four models to predict PM_2.5_ concentrations (Figure 4). A comparison between the satellite-predicted and ground-level observed values for the linear regression model, the quadratic regression model, the power regression model, and the logarithmic regression model revealed *R* values of 0.64, 0.63, 0.62 and 0.57, and RMSE values of 22.68 μg/m^3^, 24.21 μg/m^3^, 23.91 μg/m^3^, and 21.76 μg/m^3^, respectively. In addition, the slopes of the regression (*i.e.*, 0.35, 0.36, 0.36, and 0.28) suggested that all models underestimated the PM_2.5_ concentrations compared with the ground-level PM_2.5_ concentrations. These results show that the four models were good for predicting lower PM_2.5_ levels using only PARASOL level 2 AOD, which is important, especially in rural and remote areas. However, they cannot currently be used to accurately predict higher PM_2.5_ levels, which may change if additional information is incorporated into the models.

### 3.4. Models’ Performance at Different PM_2.5_ Concentrations According to the AQI

Some studies have pointed out that satellite-based PM_2.5_ models overestimate under clear weather conditions and underestimate during heavy pollution events [21,45,46]. However, few studies have analyzed the models at different ground-level PM_2.5_ concentrations. Therefore, in order to compare the four models at different ground-level PM_2.5_ values, the entire dataset was split into six sub-datasets according to the AQI (Table 2). Subsequently, the linear regression model, the quadratic regression model, the power regression model and the logarithmic regression model were developed using the six sub-datasets. It was noticed that a low level of statistical significance between the four models was found when the PM_2.5_ concentrations were greater than 75 μg/m^3^ based on the *p*-value (significance, *p* ≥ 0.05) and the *R* value, whereas the maximum *R* value was 0.44 (for the power regression model and the logarithmic regression model), and the minimum *R* value was 0.28 (for the power regression model and the logarithmic regression model) when the PM_2.5_ concentrations were less than 75 μg/m^3^. One possible reason for these results is that when air quality is moderate or severe, atmospheric composition may be the compound of fine-mode aerosols and coarse-mode aerosols. However, PARASOL official products can only provide fine-mode AOD, while can’t provide total AOD, Therefore PARASOL AOD may not directly represent the real air quality.

**Table 2:** Comparison of the four models based on the different criteria of the AQI.

AQI	PM_2.5_ (μg/m^3^)	*N*	The Linear Model		The Quadratic Model		The Power Model		The Logarithmic Model	
			*R*	*p*	*R*	*p*	*R*	*p*	*R*	*p*
0–50	0–35	1777	0.37	0.0	0.43	0.0	0.44	0.0	0.44	0.0
51–100	35–75	1636	0.29	0.0	0.30	0.0	0.28	0.0	0.28	0.0
101–150	75–115	532	0.07	0.10	0.08	0.20	0.06	0.0	0.06	0.16
151–200	115–150	176	0.11	0.13	0.11	0.31	0.10	0.0	0.10	0.18
201–300	150–250	159	0.05	0.54	0.06	0.71	0.06	0.0	0.05	0.48
>300	>250	59	0.12	0.78	0.21	0.66	0.08	0.72	0.08	0.71

## 4. Discussion

Different from most reported studies that have focused on developing empirical PM_2.5_ estimation models using MODIS AOD [17,18,19,20,21], in this study, four empirical PM_2.5_ estimation models were developed and compared based on the PARASOL level 2 AOD in China measured from 18 January 2013 to 10 October 2013. With the linear regression, the quadratic regression, the power regression and the logarithmic regression models, we obtained *R* values of 0.47, 0.48, 0.48 and 0.44 for model development, and a fairly good linear fit with *R* values of 0.64, 0.63, 0.62, and 0.57, respectively, when the models were cross validated against ground-level observed values. Moreover, the research on the four empirical models between the six levels of PM_2.5_ concentrations classified according to the AQI showed that a low level of statistical significance was found when the PM_2.5_ concentrations were greater than 75 μg/m^3^, whereas the maximum *R* was 0.44 (for the logarithmic regression model and the power model), while the minimum *R* was 0.28 (for the logarithmic regression model and the power model) when the PM_2.5_ concentrations were less than 75 μg/m^3^.

Several factors may be responsible for the models’ accuracy. One possible reason is the relatively coarse spatial resolution of the PARASOL level 2 AOD data (18.5 × 18.5 km^2^). Hu *et al.* [47] and Chudnovsky *et al.* [6] pointed out that higher spatial resolution MODIS AOD data are more suitable for estimating PM_2.5_ concentrations because they can provide more information, which helps us to understand the local environment of population exposure (e.g., business, industrial, schools, and residential areas). Another possible reason is that the kind of averaging, such as area-weighted average, or a straight average, was used for the cases where multiple ground monitoring stations existed within one PARASOL pixel may affected the models’ accuracy. However, in this paper, a straight average was used for the case where multiple ground monitoring stations existed within one PARASOL pixel. In addition, these are some outliers with the ground-level PM_2.5_ concentrations greater than 500 µg/m^3^ (Figure 2 and Figure 4) which affected the four models’ accuracy. Moreover, many previous studies have shown that models’ accuracy can be significantly improved by incorporating more information, such as the vertical profile of aerosol concentrations, atmosphere relative humidity, and land use information, *etc.* [48,49,50,51,52]. Although the vertical-and-relative humidity correcting method does not work well for PARASOL AOD, we can incorporate more information to develop statistical PM_2.5_ estimation models, such as artificial neural network model [12], GAM model (generalized additive model) [50], GWR (geographically weighted regression) model [47], mixed effects model [6], *etc.* However, we only used PARASOL level 2 AOD data, which is one of the reasons for the models’ accuracy.

It should be mentioned that although PARASOL level 2 AOD can be used to predict PM_2.5_ concentrations, and the four empirical models show a fairly good result, there are still two aspects of our method that can be improved in the future. First, we will develop PARASOL AOD inversion algorithms in order to obtain high resolution PARASOL AOD data, and determine whether the high resolution PARASOL AOD data can improve our ability to predict PM_2.5_ concentrations. Second, more information (aerosol vertical profiles, atmosphere relative humidity, wind speed, and land use information, *etc.*) will be incorporated to develop the statistical models and improve the accuracy of PARASOL satellite prediction.

## 5. Conclusions

In this study, we examined the performance of only using PARASOL level 2 AOD to predict ground-level PM_2.5_ concentrations in China. The linear regression model, the quadratic regression model, the power regression model and the logarithmic regression model were developed using PARASOL level 2 AOD and ground-level PM_2.5_ concentrations. The results show that the four empirical models show fairly good results, and underestimate the PM_2.5_ concentrations compared with the ground-level PM_2.5_ concentrations. In addition, after all the data were classified into six levels according to the AQI, a low level of statistical significance between the four empirical models was found when the ground-level PM_2.5_ concentrations were greater than 75 μg/m^3^. This study will provide useful information for estimating ground-level PM_2.5_ concentrations using PARASOL AOD, and the accuracy of the models would be improved by incorporating more parameters into the models.

## Abbreviations

The following abbreviations are used in this manuscript:

### Abbreviations

PM_2.5_: Particles with an aerodynamic diameter ≤2.5 μm
MODIS: Moderate Resolution Imaging Spectroradiometer
MISR: Multi Angle Imaging Spectroradiometer
SeaWiFS: Sea viewing Wide Field of view Sensor
GEOS: Goddard Earth Observing System
PARASOL: Polarization & Anisotropy of Reflectances for Atmospheric Sciences coupled with Observations from a Lidar
POLDER: Polarization and Directionality of Earth’s Reflectances
AOD: Aerosol optical depth
R: correlation coefficient
AQI: Air Quality Index
CNEMC: China National Environmental Monitoring Center
IGBP: International Geosphere Biosphere Programme
